# Ubiquitin‐specific protease 19 blunts pathological cardiac hypertrophy via inhibition of the TAK1‐dependent pathway

**DOI:** 10.1111/jcmm.15724

**Published:** 2020-08-14

**Authors:** Rujia Miao, Yao Lu, Xue He, Xuelian Liu, Zhiheng Chen, Jiangang Wang

**Affiliations:** ^1^ Health Management Center The Third Xiangya Hospital Central South University Changsha China; ^2^ Department of Clinical Pharmacology The Third Xiangya Hospital Central South University Changsha China

**Keywords:** cardiac hypertrophy, signal transduction, ubiquitin‐specific protease

## Abstract

Ubiquitin‐specific protease 19 (USP19) belongs to USP family and is involved in promoting skeletal muscle atrophy. Although USP19 is expressed in the heart, the role of USP19 in the heart disease remains unknown. The present study provides in vivo and in vitro data to reveal the role of USP19 in preventing pathological cardiac hypertrophy. We generated USP19‐knockout mice and isolated neonatal rat cardiomyocytes (NRCMs) that overexpressed or were deficient in USP19 to investigate the effect of USP19 on transverse aortic constriction (TAC) or phenylephrine (PE)‐mediated cardiac hypertrophy. Echocardiography, pathological and molecular analysis were used to determine the extent of cardiac hypertrophy, fibrosis, dysfunction and inflammation. USP19 expression was markedly increased in rodent hypertrophic heart or cardiomyocytes underwent TAC or PE culturing, the increase was mediated by the reduction of Seven In Absentia Homolog‐2. The extent of TAC‐induced cardiac hypertrophy, fibrosis, dysfunction and inflammation in USP19‐knockout mice was exacerbated. Consistently, gain‐of‐function and loss‐of‐function approaches that involved USP19 in cardiomyocytes suggested that the down‐regulation of USP19 promoted the hypertrophic phenotype, while the up‐regulation of USP19 improved the worsened phenotype. Mechanistically, the USP19‐elicited cardiac hypertrophy improvement was attributed to the abrogation of the transforming growth factor beta‐activated kinase 1 (TAK1)‐p38/JNK1/2 transduction. Furthermore, the inhibition of TAK1 abolished the aggravated hypertrophy induced by the loss of USP19. In conclusion, the present study revealed that USP19 and the downstream of TAK1‐p38/JNK1/2 signalling pathway might be a potential target to attenuate pathological cardiac hypertrophy.

## 1 BACKGROUND

Cardiac hypertrophy can be induced by pressure/volume overload, hormonal factors and ischaemia injury, and is an initially adaptive process to these stimuli.^1^ As the hypertrophic course progresses, the heart becomes pathologically remodelled: the entire heart continuously enlarges, the volume of each myocardial cell, the inflammatory response is triggered, and the production of extracellular matrix proteins sharply increases with the reactivation of foetal gene expression, ultimately, leading to cardiac dysfunction and even heart failure.^2^ The development of cardiac pathological hypertrophy involves a complex process of myocyte molecular modification and the excessive activation of multiple signalling cascades such as Akt, extracellular signal‐regulated kinase 1/2 (ERK1/2), and transforming growth factor beta‐activated kinase 1 (TAK1)‐p38/ c‐JUN (protein encoded by JUN gene) N‐terminal kinase 1/2 (JNK1/2). The identification of novel molecules and related pathways within the hypertrophic signalling network may potentially provide a therapeutic target for the prevention of myocardial hypertrophy and dysfunction.

Ubiquitin‐specific protease 19 (USP19) is a 150‐kDa enzyme in the ubiquitin‐specific protease family, which is the largest family of deubiquitinating enzymes. USP19 contains a carboxy‐terminal located core catalytic domain delimited by cysteine, histidine, boxes, surrounding sequences separating these boxes are domains that mediate substrate specificity, protein interactions and subcellular location.^3^ USP19 is involved in various cellular processes, which include endoplasmic reticulum‐associated degradation,^4^ misfolding‐associated protein secretion,^5^ immune response,^6^ cell growth^7^ and adipogenesis.^8^ Importantly, USP19 has been extensively documented as an atrophy‐promoting gene in skeletal muscles^9^, ^10^ through the function of down‐regulating muscle myoblast differentiation,^11^ increasing protein degradation^12^ and decreasing protein synthesis.^13^ In particular, depletion of USP19 in muscle myoblast increases the expression level of myogenin, which is a myogenic transcriptional factor, and this inhibits the oestradiol‐induced repression of myogenesis and promotes the synthesis of myofibrillar proteins.^14^ Furthermore, the silencing USP19 inhibits denervation‐ or glucocorticoid‐induced muscle atrophy by suppressing protein degradation, with a notably decreased expression of the ubiquitin ligases MuRF1 and MAFbx/atrogin‐1,^12^ which are correlated to the cardiac hypertrophy blockade.^15^, ^16^ Moreover, USP19‐knockout mice in response to fasting manifested increased rates of muscle protein synthesis.^13^ Given the similarity between skeletal muscle cells and cardiomyocytes, and that USP19 is expressed in rodent hearts in considerable levels,^17^ it can be speculated that USP19 may protect against cardiac hypertrophy. Moreover, USP19 interacts with and inhibits the activation of TAK1.^18^ Thus, the evidence that TAK1 participates in mediating cardiac hypertrophy^19^ enhances the possibility of the cardioprotective effect of USP19.

In the present study, increased levels of USP19 in the heart or cardiomyocytes in response to hypertrophic stimuli were observed, and the increase was partially mediated by the reduction of Seven In Absentia Homolog‐2 (SIAH2). By subjecting USP19 knockout mice to angiotensin II (AngII) stimuli or cardiac pressure overload, which was performed by transverse aortic constriction (TAC), the USP19 deficiency exacerbated the cardiac hypertrophy, fibrosis, dysfunction and inflammation. Furthermore, the gain‐of‐function and loss‐of‐function approaches in cardiomyocytes were also used to explore the anti‐hypertrophic effect of USP19. Mechanistically, this beneficial effect was largely dependent on the suppression of the TAK1‐dependent signalling pathway.

## 2 METHODS

### 2.1 Mice and modelling

USP19 knockout mice on the C57BL/6 background were generated using the CRISPR/Cas9 method and obtained from the A3‐Lab Animal Center (Wuhan University, Wuhan, China).^18^ The animal protocol was approved by the Animal Care and Use Committee of Third Xiangya Hospital, Central South University, China. The investigation complied to the National Institutes of Health (NIH) Guide for the Care and Use of Laboratory Animals.

Male C57/B6 mice (8‐10 weeks old, weighting 25.5‐27.0 g) were subjected to a sham or TAC operation. These mice were anaesthetized with an intraperitoneal injection of pentobarbital sodium (90 mg/kg; Sigma, St. Louis). After ensuring a calm breath and the absence of toe‐pinch reflex, these mice were placed on a heating pad in the supine position. Next, the skin was opened at the midline position of the thoracic vertebra and thoracic clavicle, and the connective tissue and the muscle layer were separated by forceps. After pulling the thyroid gland aside, the aorta arch was exposed, aortic constriction was performed using 7‐0 silk sutures by banding the aorta arch with a 26‐G needle, and the needle was quickly removed. In contrast, sham‐operated mice underwent the same procedure without aorta constriction. Afterwards, Doppler echocardiography was used to confirm whether the constriction of the aorta was adequate.

The model of Ang II‐induced cardiac hypertrophy was also constructed as previously described.^19^ Angiotensin II (1.4 mg/kg/d and dissolved in 0.9% NaCl) was subcutaneously infused for 4 weeks using an osmotic minipump (Alzet model 2004; Alza Corp, Palo Alto) implanted into each mouse. The control mice group received the same procedures as the experimental animals, with the same dose of saline infusion.

### 2.2 Cultured NRCMs with recombinant adenoviral vectors and immunofluorescence/confocal microscope analysis

Neonatal Sprague‐Dawley rats were killed by decapitation in accordance with the Guides for the Care and Use of Laboratory Animals published by the US National Institutes of Health. Primary neonatal rat cardiomyocytes (NRCMs) from the ventricle were isolated and cultured in DMEM/F12(C11330, GIBCO) medium, which included 10% foetal calf serum, 1% penicillin/streptomycin and 5‐bromodeoxyuridine(0.1mM, to inhibit fibroblast proliferation) for 24 hours under the condition of 37℃ and 5% CO_2_. Then, cardiomyocytes were infected with adenoviral vectors and cultured for 12 hours, then the medium containing no serum for starvation was replaced, added 50 μmol/L of PE and/or TAK1 inhibitor NG25 (iTak1, 2.5 μmol/L, HY‐15434, MedChem Express, New Jersey) was added and cultured for another 24 hours. The control group was treated with the same amount of phosphate buffered saline (PBS) and/or dimethyl sulphoxide (DMSO). In order to overexpress USP19, the entire coding region of the mouse USP19 gene, under the control of the cytomegalovirus promoter, was inserted in replication‐defective adenoviral vectors (AdUSP19), and a similar adenoviral vector that encoded the green fluorescent protein gene was made as the control (AdGFP). In order to silence USP19, replication‐defective adenoviral vectors that harboured USP19 short hairpin RNA (AdshUSP19) were constructed, and the non‐targeting shRNA(AdshRNA) was used as the control. The sequence for the overexpression and downexpression of USP19 is as follows: AdUSP19‐mouse‐F: GGCTAGCGATATCGGATCCGCCACCATGTCTGCAGGGGCCAGTG, AdUSP19‐mouse‐R: CGTCCTTGTAATCACTAGTTCTCCAGCGACTCTGAGATACC, AdshUSP19‐rat‐F: CCGGGCCTCTCGAATCGATATCTGCCTCGAGGCAGATATCGATTCGAGAGGCTTTTTG and AdshUSP19‐rat‐R: AATTCAAAAAGCCTCTCGAATCGATATCTGCCTCGAGGCAGATATCGATTCGAGAGGC. NRCMs were infected with the corresponding adenoviruses followed by subsequent Western blotting detection and immunofluorescence analysis. We also constructed an adenovirus vector‐mediated rat SIAH2 overexpression plasmid (AdFlag‐SIAH2) and shRNA plasmid targeting rat SIAH2 to knock down SIAH2 and transfected NRCMs by standard molecular biology techniques. Primer of rat SIAH2 overexpression plasmid: F‐GGCTAGCGATATCGGATCCGCCACC ATGAGCCGCCCGTCCTC, R‐CGTCCTTGTAATCACTAGT CTGACAGCATGTAGATATTGTGAC; Rat shSIAH2 plasmid: F‐CCGGCCAATGCCGCCAGAAGTTGCTCGAGCAACTTCTGGCGGCATTGG TTTTTG, R‐AATTCAAAAACCAATGCCGCCAGAAGTTGCTCGAG CAACTTCTGGCGGCATTGG. AdFlag and AdshRNA (mature scrambled RNA sequence: CAACAAGATGAAGAGCAGGAA) were used as controls. Cells were fixed in 4% formaldehyde for 30 minutes, permeabilized with 0.1% Triton X‐100 and blocked in 10% BSA solution. Then, these cells were incubated with primary antibodies against α‐actin (1:100 dilution; 05‐384 Merck Millipore, Billerica, USA) followed by fluorescent secondary antibodies(donkey antimouse IgG, 1:200 dilution; A21202, Invitrogen, Carlsbad) and 4‐6‐diamidino‐2‐phenylindole. The cell surface areas were calculated using the Image‐Pro Plus 6.0 software. The NRCMs were also stained with USP19 (GTX87472, 1:500; Genetex, Irvine) primary antibody and corresponding secondary antibody, and images were obtained by using the confocal laser scanning microscope (TCS SP8; LEICA, Wetzlar, Germany).

### 2.3 Echocardiography measurements

Echocardiography (MyLab Gamma; Biosound Esaote Inc with an 18‐MHz probe, Genoa, Italy) measurements were performed after mice were anaesthetized with isoflurane (1.5%‐2.0%) and placed in the supine position. M‐mode tracings derived from the short axis of the left ventricle (LV) at the level of the papillary muscles were recorded. The left ventricular end‐diastolic dimension (LVEDd) and left ventricular end‐systolic dimension (LVESd) were measured, and the fractional shortening fraction (FS, %) and ejection fraction (EF, %) were calculated as previously described.^20^

### 2.4 Histological analysis

The hearts were excised at four weeks after TAC, placed in 10% KCl to keep in diastole, then fixed in 10% formalin for 48 hours and embedded in paraffin following standard histological procedures. Subsequently, these hearts were transversely cut at the apex to visualize the ventricles and sliced. Then, the 5‐μm‐thick sections chosen from the mid‐papillary muscle level of each heart were stained with haematoxylin and eosin (H&E: haematoxylin, Servicebio, Wuhan, China, G1004; eosin, BASO, New Taipei, BA‐4024) to assess the histopathology, and picrosirius red (263, Hedebio, Beijing) to assess the collagen deposition. Images of the 100 oval‐shaped myocytes and collagen deposition were captured from five samples by microscopy and measured using a quantitative digital analysis imaging system (Image‐Pro Plus6.0).

### 2.5 Quantitative real‐time polymerase chain reaction and Western blotting

According to the manufacturer's instructions, TRIzol reagent (15596‐026; Invitrogen, Carlsbad) was used to extract the RNA from LV tissues and cultured cardiomyocytes. Then, the RNA was reverse‐transcribed into cDNA using a Transcriptor First Strand cDNA Synthesis Kit (04896866001; Roche, Basle, Switzerland), and the quantitative real‐time polymerase chain reaction (PCR) amplification was performed using the SYBR Green PCR Master Mix (04887352001; Roche, Basle, Switzerland). The primers of the detected gene are as follows: 5ʹ‐TCGGAGCCTACGAAGATCCA‐3ʹ (forward, F) and 5ʹ‐TTCGGTACCGGAAGCTGTTG‐3ʹ (reverse, R) for mouse atriopeptin (ANP); 5ʹ‐GAAGGACCAAGGCCTCACAA‐3ʹ (F) and 5ʹ‐TTCAGTGCGTTACAGCCCAA‐3ʹ (R) for mouse brain natriuretic peptide (BNP); 5ʹ‐CAACCTGTCCAAGTTCCGCA‐3ʹ (F) and 5ʹ‐TACTCCTCATTCAGGCCCTTG‐3ʹ (R) for mouse myosin heavy chain 7 (Myh7); 5ʹ‐TGCTAACGTGGTTCGTGACCGT‐3ʹ (F) and 5'‐ACATCTTGAGGTCGCGGCATGT‐3' (R) for mouse collagen Iα; 5ʹ‐ACGTAAGCACTGGTGGACAG‐3ʹ (F) and 5ʹ‐CCGGCTGGAAAGAAGTCTGA‐3ʹ (R) for mouse collagen III; 5ʹ‐TGACCCCTGCGACCCACA‐3ʹ (F) and 5ʹ‐TACACCGACCCACCGAAGACACAG‐3ʹ (R) for mouse connective tissue growth factor (CTGF);

5ʹ‐GGAGATGCTAGGAGAGTGTCCC‐3ʹ (F) and 5ʹ‐GCGCAGCTTAACAATCACCTC‐3ʹ (R) for mouse USP19; 5ʹ‐CGGCAGTTCTGTTTCCCTGT‐3ʹ (F) and 5ʹ‐ACTGACCCTTGGAGTAATGGG‐3ʹ (R) for mouse SIAH2; 5ʹ‐ACTCCACTCACGGCAAATTC‐3ʹ (F) and 5ʹ‐TCTCCATGGTGGTGAAGACA‐3ʹ (R) for mouse glyceraldehyde‐3‐phosphate dehydrogenase (GAPDH); 5ʹ‐ATGGGCTCCCTCTCATCAGT‐3ʹ (F) and 5ʹ‐GCTTGGTGGTTTGCTACGAC‐3ʹ (R) for mouse Tnf‐α; 5ʹ‐GACTTCACCATGGAACCCGT‐3ʹ (F) and 5ʹ‐CAGGGAGGGAAACACACGTT‐3ʹ (R) for mouse IL‐1β; 5ʹ‐ATGACAGCACGGTGACAACA‐3ʹ (F) and 5ʹ‐CAAATCCGGGAAGCCTGG‐3ʹ (R) for rat USP19; 5ʹ‐AAAGCAAACTGAGGGCTCTGCTCG‐3ʹ (F) and 5ʹ‐TTCGGTACCGGAAGCTGTTGCA‐3ʹ (R) for rat ANP; 5ʹ‐TGCCCCAGATGATTCTGCTC‐3ʹ (F) and 5ʹ‐TGTAGGGCCTTGGTCCTTTG‐3ʹ (R) for rat BNP; 5ʹ‐TGTGAACGGATTTGGCCCTA‐3ʹ (F) and 5ʹ‐GATGGTGATGGGTTTCCCGT‐3ʹ (R) for rat GAPDH.

Western blotting was performed on the cardiac tissues or cultured cardiomyocytes in accordance with previously described methods.^21^ The information of the antibodies are listed as follows: USP19 (GTX87472, Genetex, Irvine), p‐TAK1 (4531, CST, Boston), TAK1 (5206, CST), p‐ERK1/2 (4370, CST), ERK1/2 (4695, CST), p‐JNK1/2 (4668, CST), JNK1/2 (9252, CST), p‐p38 (4511,CST), p38 (9212,CST), p‐IKKα (AP0505, Abclonal, Woburn), IKKα (2682, CST), p‐IκBα (9246, CST), IκBα (4814, CST), p‐p65 (3033, CST), p65 (8242, CST), Flag (M185, MBL, Nagoya), SIAH2 (NBP2‐93257, Novus, Littleton) and GAPDH (2118, CST). The results were expressed as the average relative expression normalized to GAPDH.

### 2.6 Statistical analysis

Continuous data were presented as the mean ± SD. The analysis between two groups was performed using two‐tailed *t* test. One‐way ANOVA followed by Bonferroni or Tamhane's T2 post hoc test was applied for comparisons among more than two groups. *P* ＜ 0.05 was considered statistically significant. The SPSS 21.0 software was applied.

## 3 RESULTS

### 3.1 The phenotype of gain and loss of function of USP19 in hypertrophic stimuli‐induced models revealed its anti‐hypertrophy effect

**FIGURE 1:**
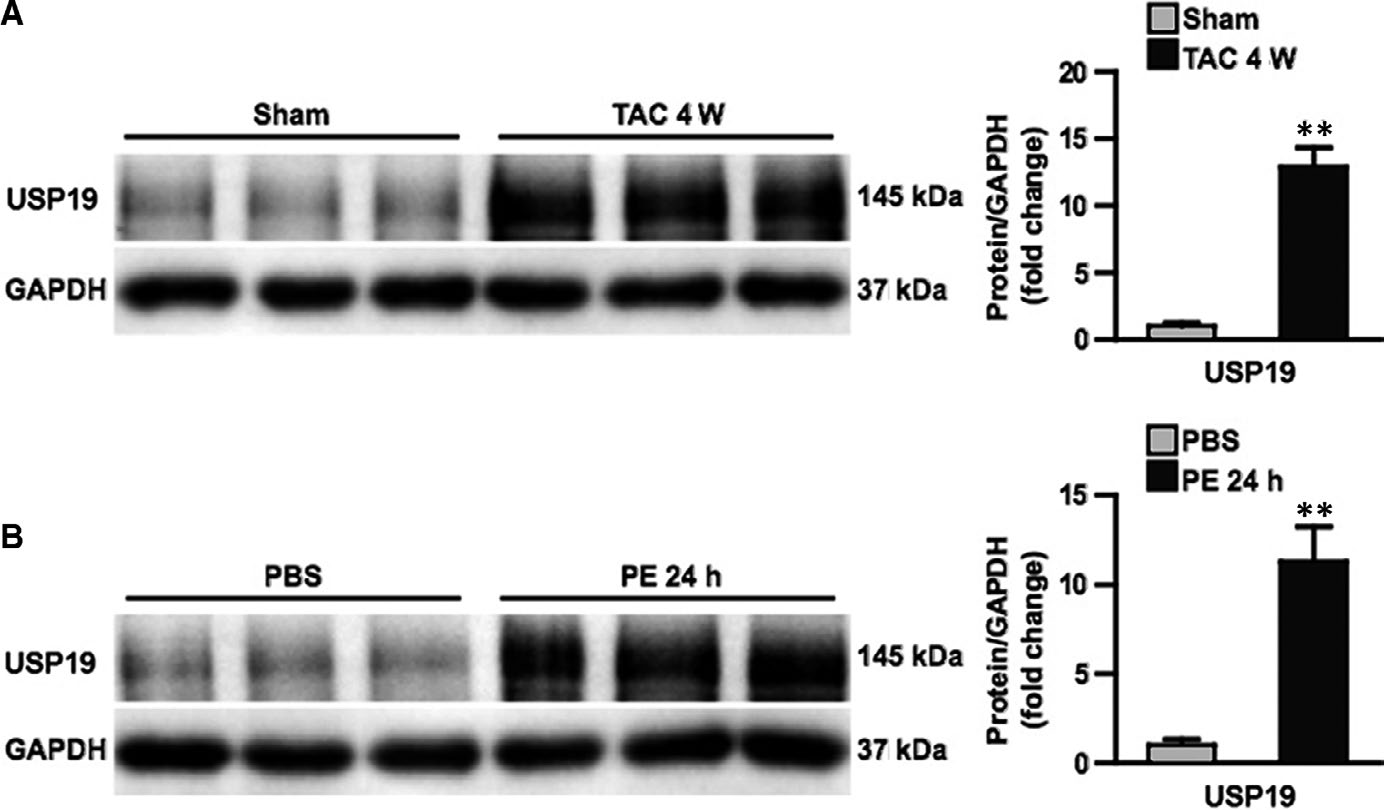
Ubiquitin‐specific protease 19 (USP19) expression is increased in murine hypertrophic hearts and cardiomyocytes. A, Left, Western blot bands of USP19 in mice subjected to sham or transverse aortic constriction (TAC) surgery at 4 wk. Right, protein expression levels were normalized to glyceraldehyde‐3‐phosphate dehydrogenase (GAPDH) and compared between indicated groups (n = 3, **P ＜ 0.01 vs sham). B, Left, Western blot analysis of USP19 in neonatal rat cardiomyocytes treated with phosphate buffered saline (PBS) or phenylephrine (PE) at 24 h. Right, protein expression levels were normalized to GAPDH and compared between indicated groups (n = 3, **P ＜ 0.01 vs PBS)

The investigators first determined the potential role of cardiac USP19 during the process of pathological hypertrophy. The intervention of both TAC and PE increased the hearts and cardiomyocytes levels of USP19 by 13 and 11 times, respectively (Figure 1A,B). Moreover, fluorescence imaging carried out with or without PE stimulation using confocal microscope revealed more abundant content of USP19 in cytoplasm under PE stimuli (Figure S4A). To explore the mechanism of USP19 elevation under stimuli stress, we first detected the levels of USP19 mRNA and found that the levels of USP19 mRNA remained unchanged before and after TAC or PE intervention (Figure S1A,B), suggesting the elevation occurred after USP19 protein had been translated. Previous study has been reported that SIAH2 is a binding partner of USP19 and promotes USP19 ubiquitylation and proteasome‐dependent degradation^22^; thus, we hypothesize USP19 may be regulated by SIAH2 in response to cardiac hypertrophy stimulation. To test this, we examined the expression of SIAH2 in the heart of mice before and after TAC surgery. The results showed that the mRNA expression level of SIAH2 was significantly decreased in the mouse heart after 4W TAC (Figure S1C). Subsequently, we up‐ or down‐regulated SIAH2 in primary cardiomyocyte using adenovirus and found that the elevated expression of USP19 protein induced by PE stimulation was blocked or exacerbated by over‐ or downexpression of SIAH2 (Figure S1C,D). These results demonstrated that SIAH2 mediated the up‐regulation of USP19 after PE stimulation.

**FIGURE 2:**
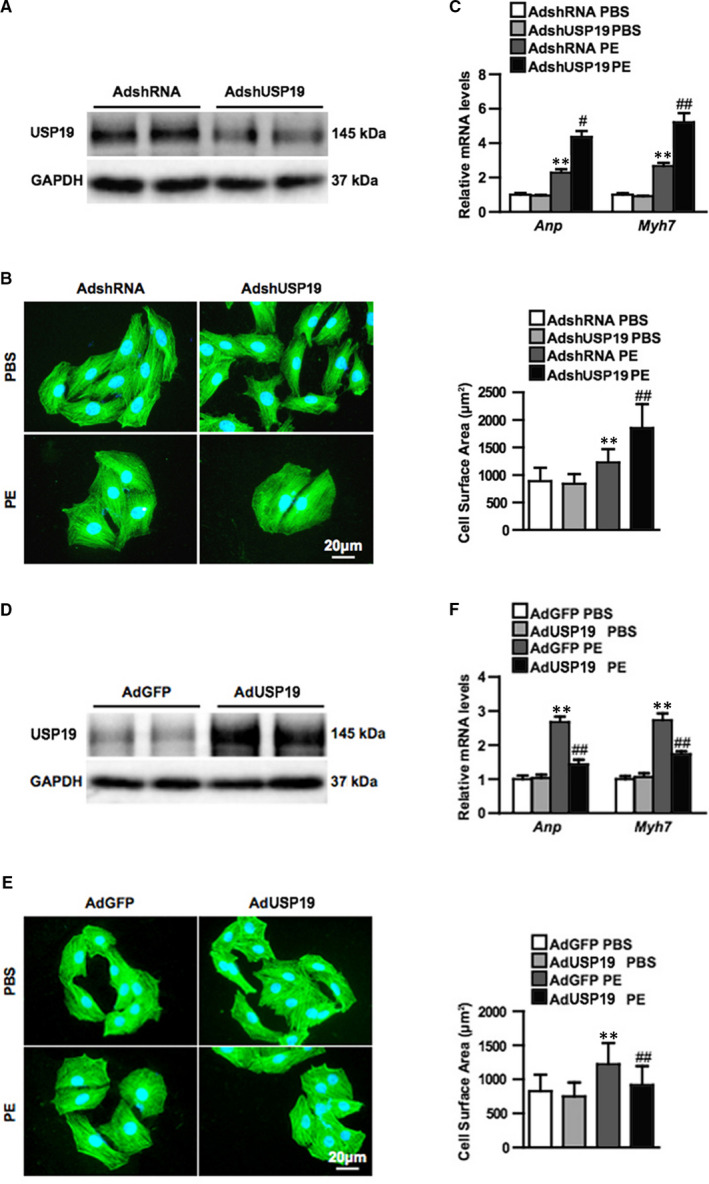
Ubiquitin‐specific protease 19 (USP19) attenuates phenylephrine (PE)‐induced cardiomyocyte hypertrophy in vitro. A, Western blot bands of USP19 levels in primary cultured cardiomyocytes infected with AdshRNA or short hairpin RNA (AdshUSP19) (n = 3 independent experiments). B, Representative microscopic images of cardiomyocytes infected with AdshRNA or AdshUSP19 and treated with phosphate buffered saline (PBS) or phenylephrine (PE). Cells were double stained of α‐actin (green) and 4‐6‐diamidino‐2‐phenylindole (blue). Scale bar, 20 μm. C, Quantitative results of the relative mRNA levels of atriopeptin (ANP) and myosin heavy chain 7 (Myh7, Above), and cross‐sectional area (Bottom) of cardiomyocytes infected with AdshRNA and AdshUSP19 in response to PBS or PE (n = 3 independent experiments, **P ＜ 0.01 vs AdshRNA/PBS, ^##^ *P* ＜ 0.01 vs AdshRNA/PE). D, Western blot bands of USP19 levels in primary cultured cardiomyocytes infected with AdGFP or AdUSP19 (n = 3 independent experiments). E, Representative microscopic images of cardiomyocytes infected with AdGFP or AdUSP19 and treated with phosphate buffered saline (PBS) or phenylephrine (PE). F, Quantitative results of the relative mRNA levels of ANP and Myh7 (Above), and cross‐sectional area (Bottom) of cardiomyocytes infected with AdGFP and AdUSP19 in response to PBS or PE (n = 3 independent experiments, **P ＜ 0.01 vs AdGFP/PBS, ^##^ *P* ＜ 0.01 vs AdGFP/PE), n.s. (no significance)

In order to explore the contribution of USP19 to the hypertrophic phenotype, NRCMs were infected with either AdshUSP19 or AdUSP19, in order to reduce or elevate the levels of USP19 in vitro (Figure 2A,D). Under basal conditions administered with PBS, neither NRCMs with AdshUSP19 nor AdUSP19 exhibited an altered size when compared with those infected with the controls of AdshRNA or AdGFP. When triggered with PE, cells in each group exhibited a hypertrophic phenotype. A comparison between the two PE groups was performed: the area of USP19‐deficient cells and AdshRNA‐infected controls increased by 1.5 and 2.2 times, respectively (Figure 2C), while NRCMs with overexpressed USP19 exhibited approximately 25.9% smaller, when compared with AdGFP‐infected cells (Figure 2F). Accordingly, the relative mRNA levels of ANP and Myh7, which are cardiomyocytes hypertrophic markers (Figure 2C,F), and the total protein levels (Figure S3A,B) detected using bicinchoninic acid (BCA) assay followed the same trend of difference among groups in terms of size value (Figure 2C,F).

**FIGURE 3:**
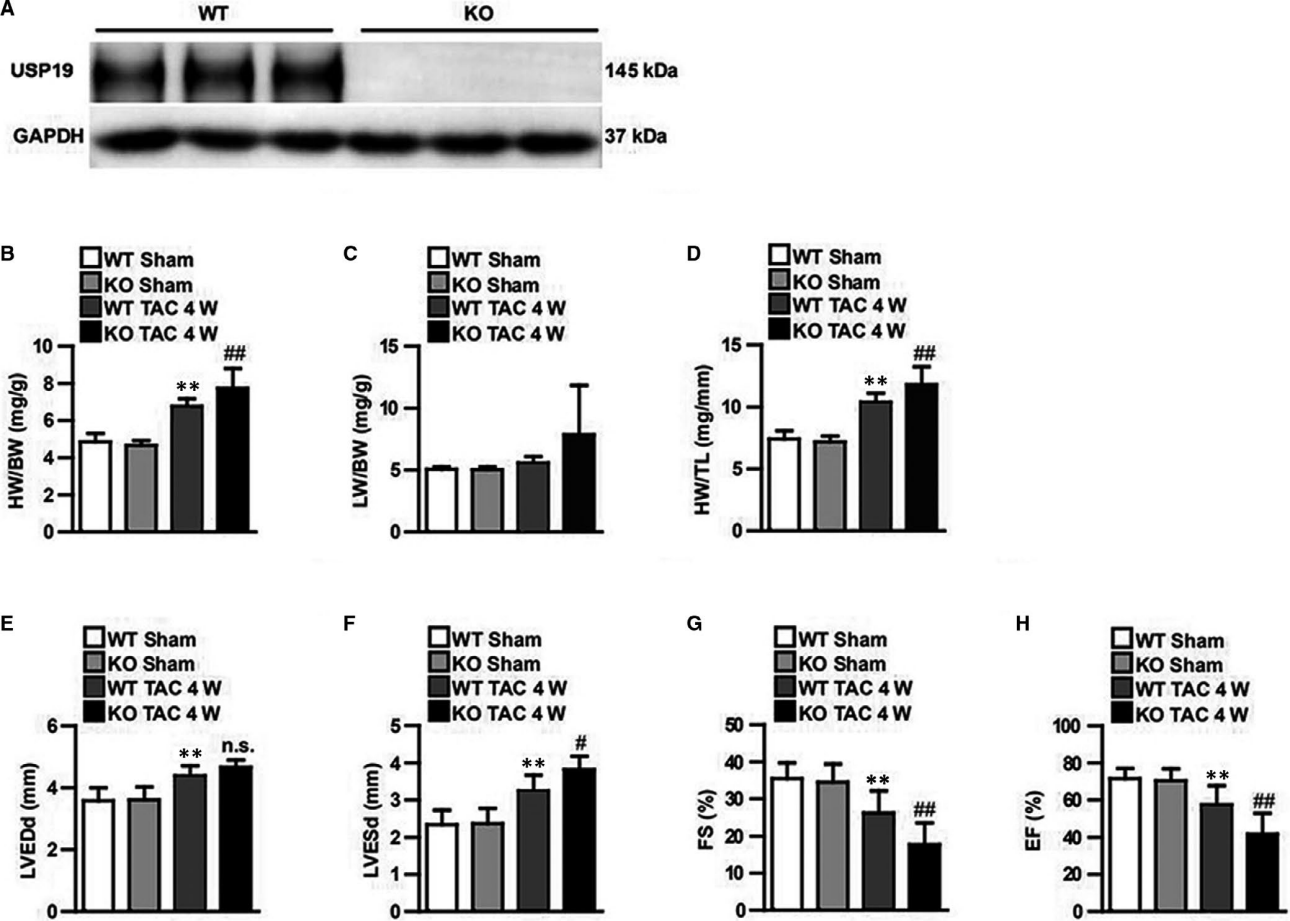
Ubiquitin‐specific protease 19 (USP19) deficiency exacerbates heart hypertrophy and dysfunction in response to transverse aortic constriction (TAC) in vivo. A, Western blot analysis revealing successful construction of USP19‐knockout (KO) mice. B‐D, Four weeks after TAC, the ratios of heart weight/bodyweight (HW/BW; left), lung weight/bodyweight (LW/BW; middle) and heart weight/tibia length (HW/TL, right) were assessed in wild‐type (WT) or KO groups subjected to sham or TAC operation (n = 10 mice per experimental group). E‐H, Echocardiographic assessment (n = 10 mice per experimental group) of left ventricular end‐diastolic diameter (LVEDd, left), left ventricular end‐systolic diameter (LVESd, middle left), fractional shortening (FS, middle right) and ejection fraction (EF, right). Data are expressed as mean ± SD. **P ＜ 0.01 or *P ＜ 0.05 vs WT/sham; ^##^ *P* ＜ 0.01 or ^#^ *P* ＜ 0.05 vs WT/TAC

**FIGURE 4:**
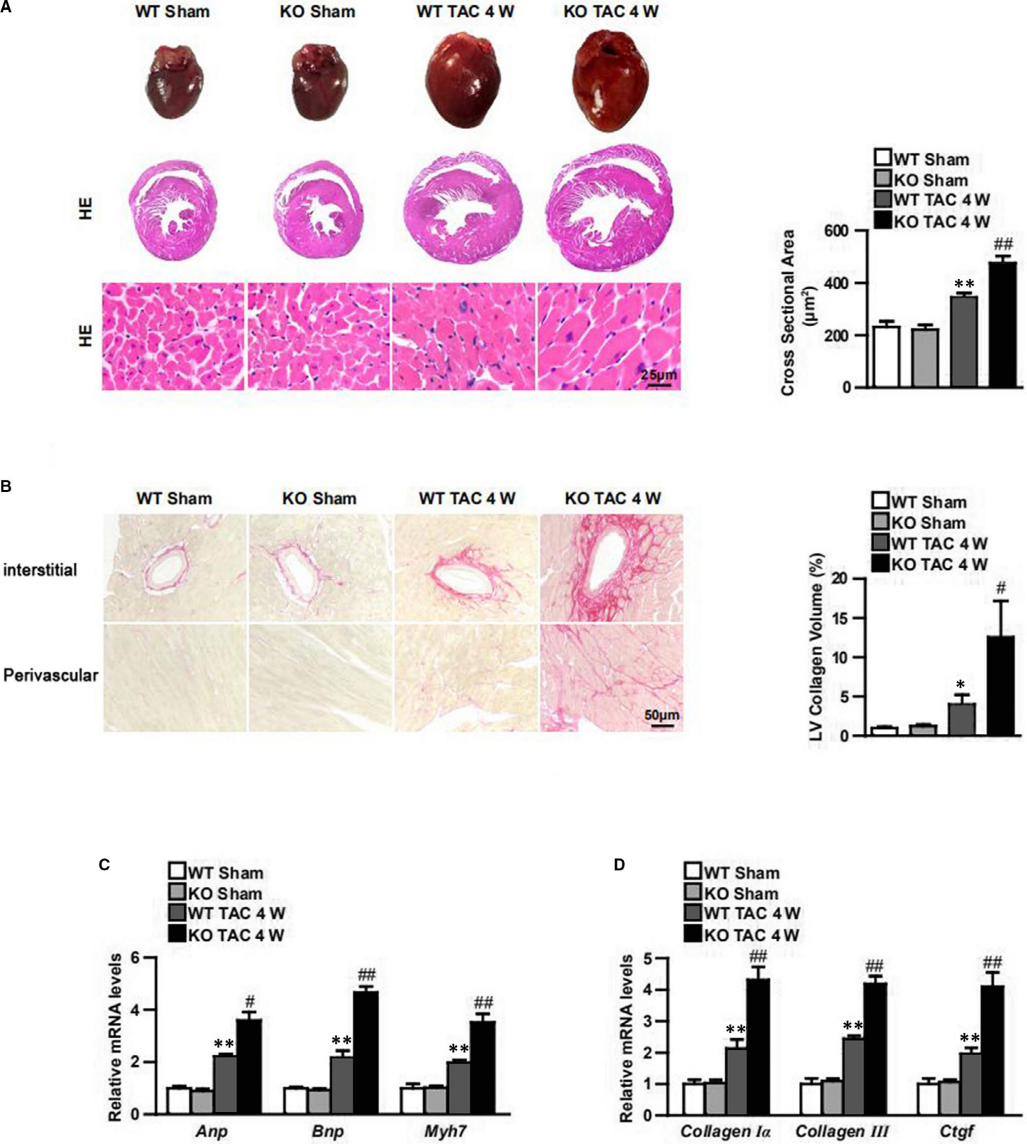
Ubiquitin‐specific protease 19 deficiency exacerbates heart hypertrophy and fibrosis in response to transverse aortic constriction (TAC) in vivo. A, Representative images of the whole heart, the maximum section of the heart and cardiomyocytes (scale bar, 25 μm for haematoxylin‐eosin [HE] staining) in indicated groups (left), statistical analysis of the cross‐sectional areas of cardiomyocytes in indicated groups (n = 120 cells per experimental groups, right). B, Representative images of the cardiac fibrosis in indicated groups (scale bar, 50 μm for picrosirius red staining, left), statistical results for fibrotic areas in different groups (n = 6, right). C, D, Real‐time quantitative polymerase chain reaction showing the mRNA levels of foetal genes (left) and fibrotic markers (right) in the heart tissues of knockout (KO) mice and wild‐type (WT) controls after sham or TAC surgery (n = 4). Data are expressed as mean ± SD. ANP, atriopeptin; BNP, brain natriuretic peptide; CTGF, connective tissue growth factor. **P ＜ 0.01 or *P ＜ 0.05 vs WT/sham; ^##^ *P* ＜ 0.01 or ^#^ *P* ＜ 0.05 vs WT/TAC

Knockout mice were generated to further confirm the function of USP19 in vivo (Figure 3A). No pathological abnormalities in cardiac morphology or function occurred at the baseline in either USP19‐knockout mice or their wild‐type (WT) littermates. Using the 4‐week TAC method to trigger the heart remodelling, a more severe overall hypertrophic phenotype was observed in knockout mice when compared to the WT groups, as manifested by increased ratios of heart weight/bodyweight (HW/BW), lung weight/BW (LW/BW) and HW/tibia length (HW/TL) (Figure 3B‐D), and this was in parallel with the parameters of cardiac function, which included LVEDd, LVESd, FS% and EF% (Figure 3E‐H). This indicates that myocardial contraction in overload pressure‐treated USP19‐knockout mice worsened when compared to the WT group. Furthermore, an enlarged gross size of the heart and cardiomyocytes was observed in the TAC‐induced groups, among which USP19‐knockout mice exhibited an even larger heart (Figure 4A). Subsequently, it was evaluated that the effects of the fibrotic phenotype, perivascular and interstitial fibrosis were notably increased in groups subjected in TAC, and fibrosis was triggered more in USP19‐deleted mice (Figure 4B). Furthermore, by detecting the expression of hypertrophic (ANP, BNP and Myh7) and fibrotic (collagen Ia, collagen III and CTGF) markers, the hypertrophic and fibrotic differences among the groups were reconfirmed in accordance (Figure 4C,D). Considering that aroused inflammatory response was one of the phenotypes of cardiac hypertrophy, we subsequently detected total and phosphorylated IKKα, IκBα and p65 in NF‐κB axis, and transcriptional levels of pro‐inflammatory cytokines in WT and USP19‐KO mice with or without TAC intervention. TAC‐treated mice displayed higher mRNA levels of interleukin‐1β (IL‐1β), tumour necrosis factor α (TNF‐α), and more phosphorylated IKKα, IκBα and p65 proteins compared to sham operation groups; and USP19 removal from heart enhanced all these levels when compared to the TAC‐stimulated WT group (Figure S4B,C). Furthermore, AngII infusion was also used to detect hormonal stimuli‐induced hypertrophic phenotype. As shown in Figure S2, 4 weeks of AngII infusion in WT mice led to significantly increased HW/BW, LW/BW, HW/TL ratios compared with saline controls (Figure S2A‐C), as well as impaired contractile function (LVEDd, LVEsd, FS%, EF%; Figure S2D‐G), enlarged myocytes (Figure S2H) and exacerbated fibrosis (Figure S2I). The structural and functional remodelling of the hearts induced by AngII was significantly exacerbated by USP19 depletion. These data indicate that USP19 represents an essential mediator of the pathological cardiac hypertrophy induced by both pressure overload and hormonal stresses.

### 3.2 USP19 suppressed cardiac hypertrophy via blocking TAK1‐dependent pathway

**FIGURE 5:**
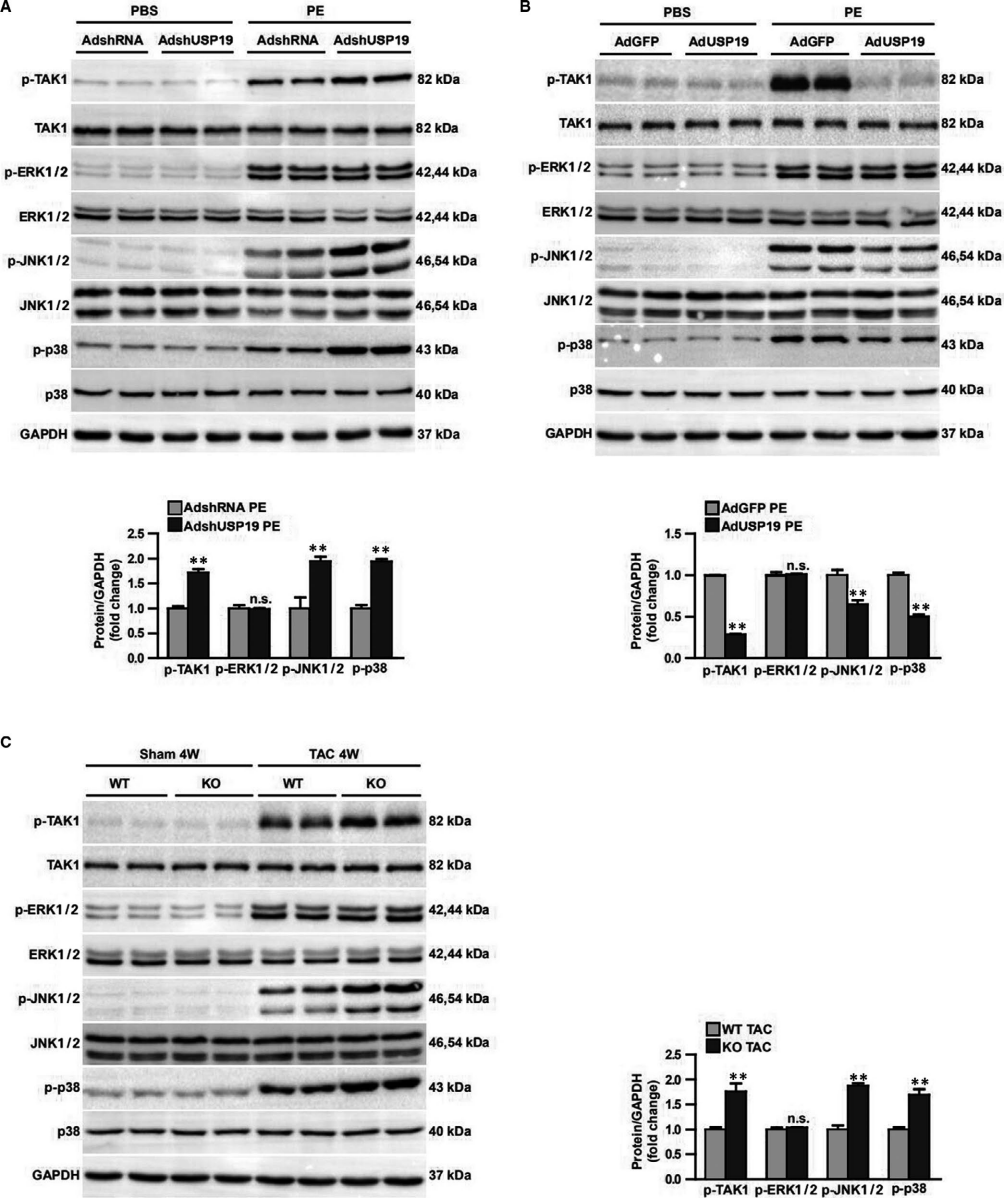
Ubiquitin‐specific protease 19 (USP19) reduces TAK1‐p38/JNK1/2 phosphorylation levels in hypertrophic hearts and cardiomyocytes. A, Above, Western blots showing the phosphorylation and total protein levels of TAK1, ERK1/2, JNK1/2 and p38 in primary neonatal rat cardiomyocytes (NRCMs) infected with AdshRNA or short hairpin RNA (AdshUSP19) followed by treatment with phosphate buffered saline (PBS) or phenylephrine (PE). Bottom, Statistical analysis of the phosphorylation levels of TAK1, ERK1/2, JNK1/2 and p38 in PE‐treated AdshRNA and AdshUSP19 groups. n = 6, **P ＜ 0.01 vs AdshRNA/PE. B, Above, Western blots showing the phosphorylation and total protein levels of TAK1, ERK1/2, JNK1/2 and p38 in NRCMs infected with AdGFP or AdUSP19 followed by treatment with PBS or PE. Bottom, Statistical analysis of the phosphorylation levels of TAK1, ERK1/2, JNK1/2 and p38 in PE‐treated AdGFP and AdUSP19 groups. n = 6, **P ＜ 0.01 vs AdGFP/PE. C, Above, Western blots showing the phosphorylation and total protein levels of TAK1, ERK1/2, JNK1/2 and p38 in wild‐type (WT) or USP19‐knockout (KO) mice subjected to sham or transverse aortic constriction (TAC) surgery. Bottom, densitometry analyses of the phosphorylation levels of TAK1, ERK1/2, JNK1/2 and p38 in TAC‐treated WT and KO mice. n = 6, **P ＜ 0.01 vs WT/TAC. n.s., no significance

**FIGURE 6:**
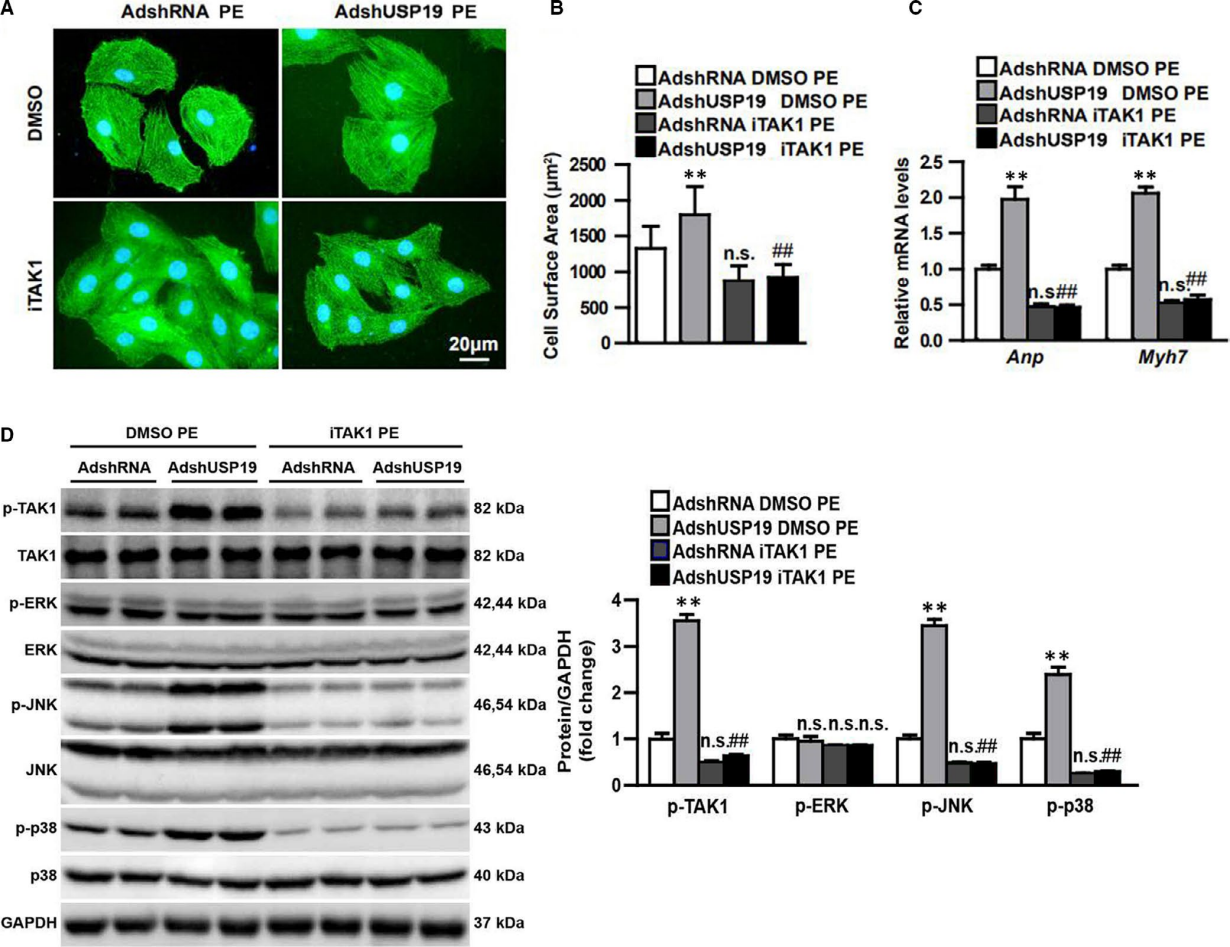
Inhibition of the TAK1 blunts the hypertrophic effect of ubiquitin‐specific protease 19 (USP19) deficiency in cardiomyocytes. A, B, Left, representative microscopic images of phenylephrine (PE)‐treated NRCMs infected with AdshRNA or AdshUSP19 in indicated groups, scale bar, 20 μm. Right, iTAK1 decreased cross‐sectional size of NRCMs to similar extents in AdshRNA and AdshUSP19 mice (n = 40 cells per experimental groups). C, Quantitative results of the relative mRNA levels of atriopeptin (ANP) and myosin heavy chain 7 (Myh7) in PE‐treated groups (n = 3 independent experiments). D, Left, Western blot analysis revealed the levels of TAK1 in primary neonatal rat cardiomyocytes (NRCMs) infected with AdshRNA or short hairpin RNA (AdshUSP19) followed by treatment with dimethyl sulphoxide (DMSO) or TAK1 inhibitor (iTAK1). Right, statistical analysis of the phosphorylation levels of TAK1, ERK1/2, JNK1/2 and p38. **P ＜ 0.01 and n.s. (no significance) on AdshUSP19 DMSO PE, vs AdshRNA DMSO PE; ^##^ *P* ＜ 0.01 and n.s. on AdshUSP19 iTAK1 PE, vs AdshUSP19 DMSO PE; n.s, on AdshUSP19 iTAK1 PE, vs AdshUSP19 iTAK1 PE

Since the anti‐hypertrophy function of USP19 was confirmed, the investigators determined the underlying mechanism that mediated this effect. The investigators chose to detect the mitogen‐activated protein kinase signalling, including ERK1/2, p38 and JNK1/2, which are typical pathway that mediated cardiac hypertrophy. The present data suggest that the total levels of MAPK components were almost the same among the baseline groups of PBS or sham, and the intervention groups of PE or TAC. When the phosphorylation levels of ERK1/2, p38 and JNK1/2 were detected, the grey level of the baseline groups was visibly low. However, the grey value could significantly be elevated in response to the overload stimuli, and compared with the increasing rate between the WT group and USP19‐altered group, merely the phosphorylated levels of JNK1/2, p38 and their common upstream kinase TAK1, but not ERK1/2, notably increased significantly in USP19‐knockout mice, when compared to WT mice (Figure 5C). Further studies have verified the PE‐cultured cellular signalling pathway in vitro, and the USP19 removal from NRCMs enhanced the phosphorylation of TAK1, JNK1/2 and p38 when compared to the PE‐stimulated AdshRNA group, while USP19 up‐regulation conversely blocked the phosphorylated activity of the TAK1‐p38/JNK1/2 pathway in NRCMs, but the levels of ERK1/2 remained the same in the USP19‐altered groups (Figure 5A,B). In order to confirm whether TAK1 and the downstream p38/JNK1/2 signalling contributes to the effect of USP19 on cardiac hypertrophy, iTAK1 was added to PE‐cultured cells to abolish the expression of phosphorylated TAK1 (Figure 6D), it was found that iTAK1 recovered the phosphorylated p38, JNK1/2 (Figure 6D), the cell size and hypertrophic markers (ANP and Myh7) (Figure 6B,C) to low levels in AdshRNA‐infected and even AdshUSP19‐infected NRCMs when compared to the iTAK1‐negative groups, and iTAK1 decreased these index to similar levels in AdshRNA and AdshUSP19 mice.

## 4 DISCUSSION

USP19 was identified as an essential regulator of cardiac remodelling. The data demonstrated that USP19 protein expression notably increased in hypertrophic hearts and cardiomyocytes. Furthermore, USP19 deletion accelerated the hypertrophic response to hormonal stimuli/pressure overload in mouse hearts. Consistently, in vitro experiments suggested the role of USP19 in inhibiting NRCMs hypertrophy. The USP19‐mediated cardioprotection was attributed to the blocking of the TAK1‐p38/JNK1/2‐dependent pathway, which was confirmed by the inhibition of TAK1. To our knowledge, the present study is the first evidence for the vital function of USP19 in protecting against pathological cardiac hypertrophy.

In the present study, a significantly increase in USP19 expression during the development of pathological cardiac hypertrophy increased the possibility that USP19 may play a critical role in this processes. USP19 mRNA levels remaining unchanged with or without hypertrophic stimuli suggested that post‐translational modification should be responsible for USP19 elevation. SIAH2 binds to USP19 and promotes USP19 ubiquitylation and proteasome‐dependent degradation^22^; then, the hypothesis of a causal link between SIAH2 and USP19 in cardiomyocyte hypertrophy was established: Decreased transcription of SIAH2 during hypertrophic progress led to less degradation of USP19, and this mechanism partially accounted for USP19 elevation. Other unknown mechanisms might still exist in mediating the decrease of USP19 protein and remain to be explored.

It has been previously proven that the members of the ubiquitin‐specific protease family (USP18,^23^ USP15,^24^ USP4^17^ and USP14^25^) exert an effect on cardiac or cardiomyocyte hypertrophy under the stimuli of pressure overload or neurohormonal factors. However, as an extensively accepted enzymes that modulated the key processes that control the skeleton muscle mass,^26^ the role that USP19 might play an essential part in the myocardium remains unknown. The present study did ascertain through the data that USP19 positively prevented the pressure overload and neurohormonal factor‐induced cardiac hypertrophy. Then, the investigators chose to detect the total and phosphorylated MAPK signalling that were typically modulating the hypertrophic phenotype, as well as TAK1, which was connected to both USP19 and MAPK. USP19 attenuated the inflammatory response in vivo by interacting with TAK1 through cleaved K63 and K27‐linked polyubiquitin chains from TAK1 in a TNF‐α and IL‐1β‐dependent manner. This subsequently resulted in the impairment of TAK1 phosphorylation and the disruption of TAK1 complex, terminating the activation of the downstream inflammatory genes of TAK1.^19^ This mechanism might explain why TAK1 was involved in the USP‐19 alleviated cardiac hypertrophy. TAK1 was identified as a member of the MAPKKK family in response to transforming growth factor‐β, and under physiological circumstances, sufficient levels of TAK1 were indispensable for myocytes survival,^27^ while numerous studies including ours confirmed myocardial TAK1 resulted in chamber enlargement and heart remodelling.^17^, ^20^, ^28^, ^29^ However, the exacerbated function of TAK1 on cardiac hypertrophy was challenged by the study demonstrating that inducible expression of TAK1 protected from cardiac remodelling under pressure overload or myocardial infarction through inhibition of cardiac cell death.^30^ Such discrepancy may be reconciled by the dual role of TAK1 in the regulation of myocyte survival and hypertrophy. The combined evidence indicates that TAK1 represents a sensitive central molecular node in the heart that requires delicate regulation to maintain cardiac homeostasis. p38 and JNK1/2 are collectively called stress‐activated MAPKs and downstream molecules of TAK1.^31^ Once stimulated, the phosphorylated TAK1 transduces the signal to P38 and JNK1/2, and both of which are phosphorylated and activated.^32^ Blocking the JNK1/2 and p38 signalling pathway with a pharmacological agent delays the transition towards heart failure,^33^, ^34^ while the over‐activation of p38 and JNK1/2 induced cardiac dilation and dysfunction.^35^, ^36^ By the way, since USP19 inhibits NF‐κB activation by deubiquitinating TAK1 in immune cells,^18^ and NF‐κB and subsequent transcription of pro‐inflammatory cytokines^37^ exert inflammatory response on promoting cardiac hypertrophy^38^, ^39^, ^40^ at later stage, we speculated and confirmed that besides affecting cardiac hypertrophy, fibrosis and dysfunction, USP19 lacking in hearts might also affect the inflammatory phenotype manifested as activation of NF‐κB and more generation of pro‐inflammatory cytokines.

Cardiac fibrosis is another feature of pathological hypertrophy. Under circulatory overloaded pressure, increasing collagens and other extracellular matrix components generated by fibroblasts accumulate in the myocardium.^41^ The knockout of USP19 dramatically increased CTGF, interstitial and perivascular fibrosis. This was plausible, because TAK1‐JNK1/2 and TAK1‐p38 served as an important axis in the fibrotic response by promoting the generation of profibrotic factors including CTGF.^42^, ^43^ As an important profibrotic growth factors, CTGF was induced in cardiomyocytes under hypertrophic stimuli and promoted fibrotic response via paracrine mechanism.^44^ Therefore, these lines of evidence imply that USP19 might decrease cardiac fibrosis at least via a TAK1‐p38/JNK1/2‐dependent mechanism, and the paracrine manner might participate.

In summary, the study unfolded the role of USP19 in modulating the pathological hypertrophic phenotype in the heart and cardiomyocytes through the inhibition of the TAK1‐p38/JNK1/2 signalling pathway. This finding provided the first evidence of the involvement of USP19 in cardiac hypertrophy, and dignifies significance of the USP family in the research of cardiac homeostasis. The therapeutic pharmacological activation or genetic elevation of USP19 levels might be a reasonable strategy for cardiac hypertrophy and heart failure.

## 5 CONCLUSION

The study provides in vivo and in vitro evidence that USP19 functions as a negative regulator of pathological cardiac hypertrophy by inhibiting the TAK1‐p38/JNK1/2 signalling pathway in the hypertrophic hearts and cardiomyocytes. Thus, targeting USP19 and its interaction with TAK1 may represent promising strategies for reversing cardiac hypertrophy and dysfunction.

## CONFLICT OF INTEREST

No conflict of interest, financial or otherwise, is declared by the authors.

## AUTHOR CONTRIBUTION

**Rujia Miao:** Conceptualization (equal); Funding acquisition (lead); Methodology (equal); Writing‐original draft (lead). **Yao Lu:** Data curation (equal); Investigation (equal); Methodology (equal). **Xue He:** Investigation (equal). **Xuelian Liu:** Data curation (equal); Investigation (equal); Methodology (equal); Software (equal). **Zhiheng Chen:** Data curation (equal); Formal analysis (equal); Project administration (equal); Resources (equal). **Jiangang Wang:** Conceptualization (equal); Project administration (supporting); Supervision (lead); Writing‐review & editing (lead).

## Supporting information

Fig S1

Fig S2

Fig S3

Fig S4

Supplementary Material

## Data Availability

All data models used during the study are available from the corresponding author by request.

## References

[1] Kreusser MM , Lehmann LH , Keranov S , et al. Cardiac CaM Kinase II genes delta and gamma contribute to adverse remodeling but redundantly inhibit calcineurin‐induced myocardial hypertrophy. Circulation. 2014;130:1262‐1273. 25124496 10.1161/CIRCULATIONAHA.114.006185 PMC4316667

[2] Weber KT , Janicki JS , Shroff SG , et al. Collagen remodeling of the pressure‐overloaded, hypertrophied nonhuman primate myocardium. Circ Res. 1988;62:757‐765. 2964945 10.1161/01.res.62.4.757

[3] Nakamura N , Harada K , Kato M , et al. Ubiquitin‐specific protease 19 regulates the stability of the E3 ubiquitin ligase MARCH6. Exp Cell Res. 2014;328:207‐216. 25088257 10.1016/j.yexcr.2014.07.025

[4] Hassink GC , Zhao B , Sompallae R , et al. The ER‐resident ubiquitin‐specific protease 19 participates in the UPR and rescues ERAD substrates. Embo Rep. 2009;10:755‐761. 19465887 10.1038/embor.2009.69 PMC2727442

[5] Lee J‐G , Takahama S , Zhang G , et al. Unconventional secretion of misfolded proteins promotes adaptation to proteasome dysfunction in mammalian cells. Nat Cell Biol. 2016;18:765‐776. 27295555 10.1038/ncb3372 PMC10701763

[6] Jin S , Tian S , Chen Y , et al. USP19 modulates autophagy and antiviral immune responses by deubiquitinating Beclin‐1. Embo J. 2016;35:866‐880. 26988033 10.15252/embj.201593596 PMC4972138

[7] Lu YU , Bedard N , Chevalier S , et al. Identification of distinctive patterns of USP19‐mediated growth regulation in normal and malignant cells. PLoS One. 2011;6:e15936. 21264218 10.1371/journal.pone.0015936 PMC3022023

[8] Coyne ES , Bédard N , Gong YJ , et al. The deubiquitinating enzyme USP19 modulates adipogenesis and potentiates high‐fat‐diet‐induced obesity and glucose intolerance in mice. Diabetologia. 2019;62:136‐146. 30386869 10.1007/s00125-018-4754-4

[9] Ogawa M , Kitakaze T , Harada N , et al. Female‐specific regulation of skeletal muscle mass by USP19 in young mice. J Endocrinol. 2015;225:135‐145. 25901042 10.1530/JOE-15-0128

[10] Sundaram P , Pang Z , Miao M , et al. USP19‐deubiquitinating enzyme regulates levels of major myofibrillar proteins in L6 muscle cells. Am J Physiol Endocrinol Metab. 2009;297:E1283‐E1290. 19773579 10.1152/ajpendo.00409.2009

[11] Wiles B , Miao M , Coyne E , et al. USP19 deubiquitinating enzyme inhibits muscle cell differentiation by suppressing unfolded‐protein response signaling. Mol Biol Cell. 2015;26:913‐923. 25568336 10.1091/mbc.E14-06-1129 PMC4342027

[12] Bédard N , Jammoul S , Moore T , et al. Inactivation of the ubiquitin‐specific protease 19 deubiquitinating enzyme protects against muscle wasting. Faseb J. 2015;29:3889‐3898. 26048142 10.1096/fj.15-270579

[13] Coyne ES , Bedard N , Wykes L , et al. Knockout of USP19 deubiquitinating enzyme prevents muscle wasting by modulating insulin and glucocorticoid signaling. Endocrinology. 2018;159:2966‐2977. 29901692 10.1210/en.2018-00290

[14] Ogawa M , Yamaji R , Higashimura Y , et al. 17beta‐estradiol represses myogenic differentiation by increasing ubiquitin‐specific peptidase 19 through estrogen receptor alpha. J Biol Chem. 2011;286:41455‐41465. 21971047 10.1074/jbc.M111.276824 PMC3308857

[15] Parry TL , Desai G , Schisler JC , et al. Fenofibrate unexpectedly induces cardiac hypertrophy in mice lacking MuRF1. Cardiovasc Pathol. 2016;25:127‐140. 26764147 10.1016/j.carpath.2015.09.008 PMC4754579

[16] Li H‐H , Kedar V , Zhang C , et al. Atrogin‐1/muscle atrophy F‐box inhibits calcineurin‐dependent cardiac hypertrophy by participating in an SCF ubiquitin ligase complex. J Clin Invest. 2004;114:1058‐1071. 15489953 10.1172/JCI22220 PMC522252

[17] He B , Zhao Y‐C , Gao L‐C , et al. Ubiquitin‐specific protease 4 is an endogenous negative regulator of pathological cardiac hypertrophy. Hypertension. 2016;67:1237‐1248. 27045030 10.1161/HYPERTENSIONAHA.116.07392

[18] Lei CQ , Wu X , Zhong X , et al. USP19 inhibits TNF‐alpha‐ and IL‐1beta‐triggered NF‐kappaB activation by deubiquitinating TAK1. J Immunol. 2019;203:259‐268. 31127032 10.4049/jimmunol.1900083

[19] Ji Y‐X , Zhang P , Zhang X‐J , et al. The ubiquitin E3 ligase TRAF6 exacerbates pathological cardiac hypertrophy via TAK1‐dependent signalling. Nat Commun. 2016;7:11267. 27249171 10.1038/ncomms11267 PMC4895385

[20] Li C‐Y , Zhou Q , Yang L‐C , et al. Dual‐specificity phosphatase 14 protects the heart from aortic banding‐induced cardiac hypertrophy and dysfunction through inactivation of TAK1‐P38MAPK/‐JNK1/2 signaling pathway. Basic Res Cardiol. 2016;111:19. 26891723 10.1007/s00395-016-0536-7

[21] Chen L , Huang J , Ji Y , et al. Tripartite motif 32 prevents pathological cardiac hypertrophy. Clin Sci (Lond). 2016;130:813‐828. 26884348 10.1042/CS20150619 PMC4847158

[22] Velasco K , Zhao B , Callegari S , et al. An N‐terminal SIAH‐interacting motif regulates the stability of the ubiquitin specific protease (USP)‐19. Biochem Biophys Res Commun. 2013;433:390‐395. 23500468 10.1016/j.bbrc.2013.02.094

[23] Ying X , Zhao Y , Yao T , et al. Novel protective role for ubiquitin‐specific protease 18 in pathological cardiac remodeling. Hypertension. 2016;68:1160‐1170. 27572150 10.1161/HYPERTENSIONAHA.116.07562

[24] Isumi Y , Hirata T , Saitoh H , et al. Transgenic overexpression of USP15 in the heart induces cardiac remodeling in mice. Biochem Biophys Res Commun. 2011;405:216‐221. 21219870 10.1016/j.bbrc.2011.01.012

[25] Liu N , Chai R , Liu B , et al. Ubiquitin‐specific protease 14 regulates cardiac hypertrophy progression by increasing GSK‐3beta phosphorylation. Biochem Biophys Res Commun. 2016;478:1236‐1241. 27545607 10.1016/j.bbrc.2016.08.100

[26] Wing SS . Deubiquitinating enzymes in skeletal muscle atrophy—an essential role for USP19. Int J Biochem Cell Biol. 2016;79:462‐468. 27475983 10.1016/j.biocel.2016.07.028

[27] Li L , Chen Y , Doan J , et al. Transforming growth factor beta‐activated kinase 1 signaling pathway critically regulates myocardial survival and remodeling. Circulation. 2014;130:2162‐2172. 25278099 10.1161/CIRCULATIONAHA.114.011195 PMC4302054

[28] Zhang D , Gaussin V , Taffet GE , et al. TAK1 is activated in the myocardium after pressure overload and is sufficient to provoke heart failure in transgenic mice. Nat Med. 2000;6:556‐563. 10802712 10.1038/75037

[29] Ma Z‐G , Yuan Y‐P , Zhang X , et al. C1q‐tumour necrosis factor‐related protein‐3 exacerbates cardiac hypertrophy in mice. Cardiovasc Res. 2019;115:1067‐1077. 30407523 10.1093/cvr/cvy279

[30] Li L , Chen Y , Li J , et al. TAK1 regulates myocardial response to pathological stress via NFAT, NFkappaB, and Bnip3 pathways. Sci Rep. 2015;5:16626. 26564789 10.1038/srep16626 PMC4643217

[31] Kyriakis JM , Avruch J . Mammalian mitogen‐activated protein kinase signal transduction pathways activated by stress and inflammation. Physiol Rev. 2001;81:807‐869. 11274345 10.1152/physrev.2001.81.2.807

[32] Shim JH , Xiao C , Paschal AE , et al. TAK1, but not TAB1 or TAB2, plays an essential role in multiple signaling pathways in vivo. Genes Dev. 2005;19:2668‐2681. 16260493 10.1101/gad.1360605 PMC1283960

[33] Rose BA , Force T , Wang Y . Mitogen‐activated protein kinase signaling in the heart: angels versus demons in a heart‐breaking tale. Physiol Rev. 2010;90:1507‐1546. 20959622 10.1152/physrev.00054.2009 PMC3808831

[34] Marber MS , Rose B , Wang Y . The p38 mitogen‐activated protein kinase pathway—a potential target for intervention in infarction, hypertrophy, and heart failure. J Mol Cell Cardiol. 2011;51:485‐490. 21062627 10.1016/j.yjmcc.2010.10.021 PMC3061241

[35] Liao P , Georgakopoulos D , Kovacs A , et al. The in vivo role of p38 MAP kinases in cardiac remodeling and restrictive cardiomyopathy. Proc Natl Acad Sci USA. 2001;98:12283‐12288. 11593045 10.1073/pnas.211086598 PMC59806

[36] Wang Y , Su B , Sah VP , et al. Cardiac hypertrophy induced by mitogen‐activated protein kinase kinase 7, a specific activator for c‐Jun NH2‐terminal kinase in ventricular muscle cells. J Biol Chem. 1998;273:5423‐5426. 9488659 10.1074/jbc.273.10.5423

[37] Wu H , Yu W , Meng F , et al. Polychlorinated biphenyls‐153 induces metabolic dysfunction through activation of ROS/NF‐kappaB signaling via downregulation of HNF1b. Redox Biol. 2017;12:300‐310. 28285191 10.1016/j.redox.2017.02.026 PMC5345977

[38] Zhang X , Zhang MC , Wang CT . Loss of LRRC25 accelerates pathological cardiac hypertrophy through promoting fibrosis and inflammation regulated by TGF‐beta1. Biochem Biophys Res Commun. 2018;506:137‐144. 30340835 10.1016/j.bbrc.2018.09.065

[39] Yan SH , Zhao NW , Zhu XX , et al. Benazepril inhibited the NF‐kappaB and TGF‐beta networking on LV hypertrophy in rats. Immunol Lett. 2013;152:126‐134. 23707880 10.1016/j.imlet.2013.05.005

[40] Gupta GK , Agrawal T , DelCore MG , et al. Vitamin D deficiency induces cardiac hypertrophy and inflammation in epicardial adipose tissue in hypercholesterolemic swine. Exp Mol Pathol. 2012;93:82‐90. 22537546 10.1016/j.yexmp.2012.04.006 PMC3411274

[41] Li A‐H , Liu PP , Villarreal FJ , et al. Dynamic changes in myocardial matrix and relevance to disease: translational perspectives. Circ Res. 2014;114:916‐927. 24577970 10.1161/CIRCRESAHA.114.302819

[42] Lim AKH , Nikolic‐Paterson DJ , Ma FY , et al. Role of MKK3‐p38 MAPK signalling in the development of type 2 diabetes and renal injury in obese db/db mice. Diabetologia. 2009;52:347‐358. 19066844 10.1007/s00125-008-1215-5

[43] Kita T , Hata Y , Kano K , et al. Transforming growth factor‐beta2 and connective tissue growth factor in proliferative vitreoretinal diseases: possible involvement of hyalocytes and therapeutic potential of Rho kinase inhibitor. Diabetes. 2007;56:231‐238. 17192487 10.2337/db06-0581

[44] Matsui Y , Sadoshima J . Rapid upregulation of CTGF in cardiac myocytes by hypertrophic stimuli: implication for cardiac fibrosis and hypertrophy. J Mol Cell Cardiol. 2004;37:477‐481. 15276017 10.1016/j.yjmcc.2004.05.012

