# Supplementary figures and images for: Ubiquitin‐specific protease 19 blunts pathological cardiac hypertrophy via inhibition of the TAK1‐dependent pathway
Source: J Cell Mol Med. 2020 Aug 14;24(18):10946–57. doi: 10.1111/jcmm.15724 (PMC7521154; doi:10.1111/jcmm.15724)

**A**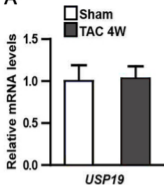**B**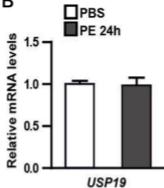**C**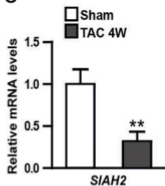**D**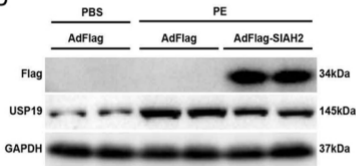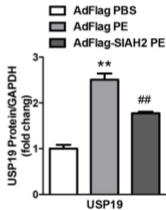**E**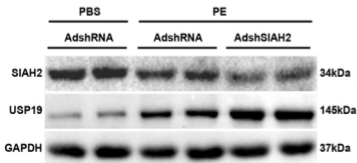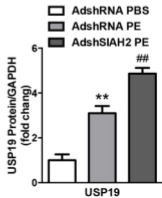

Supplement: Supplementary file 1 — Fig S1 [file JCMM-24-10946-s001.pdf]

**A**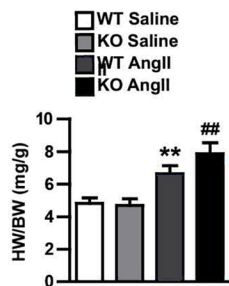**B**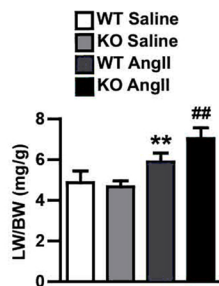**C**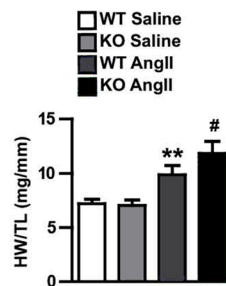**D**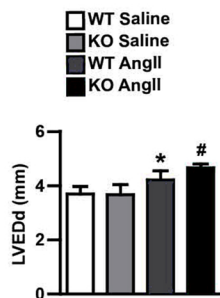**E**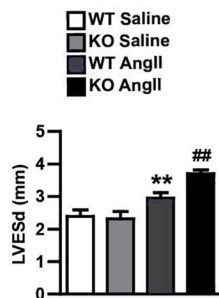**F**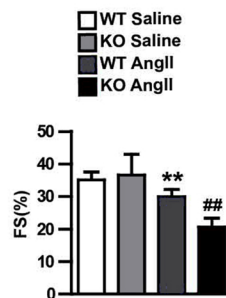**G**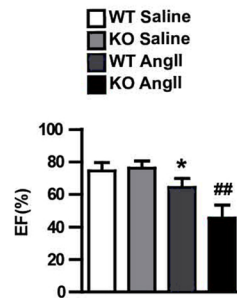**H**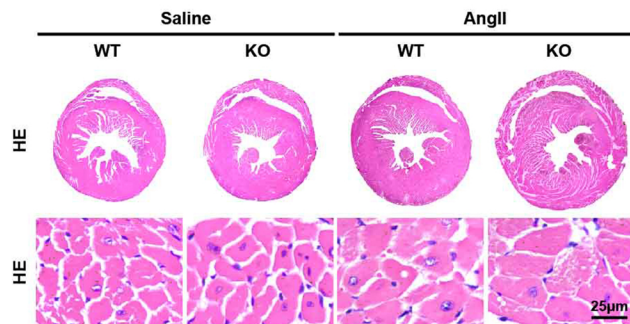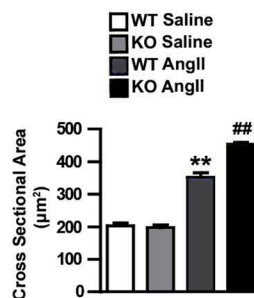**I**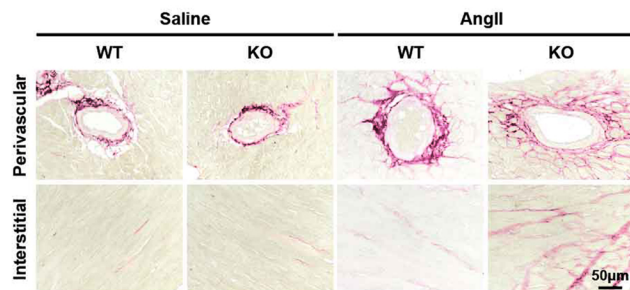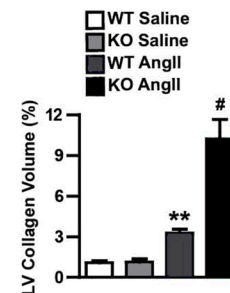

Supplement: Supplementary file 2 — Fig S2 [file JCMM-24-10946-s002.pdf]

**A**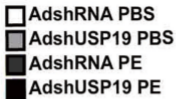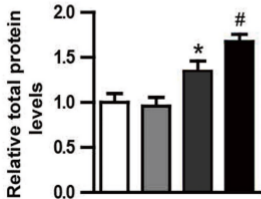**B**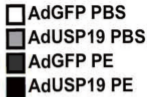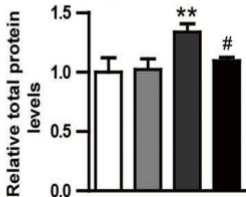

Supplement: Supplementary file 3 — Fig S3 [file JCMM-24-10946-s003.pdf]

A

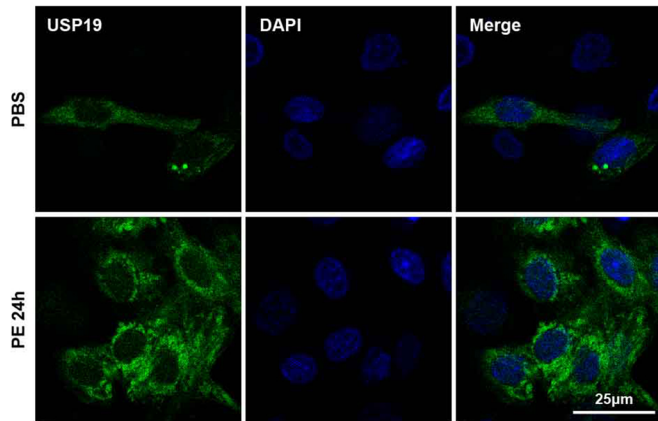

B

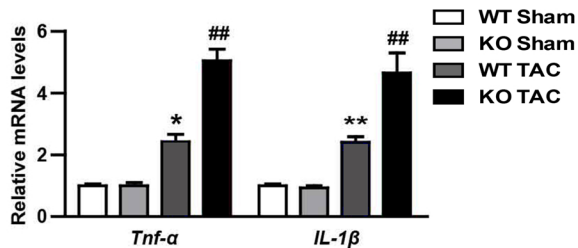

C

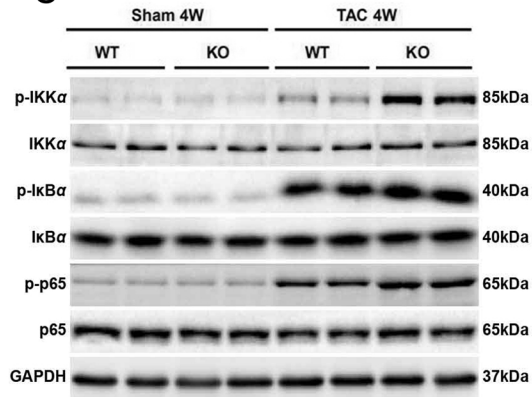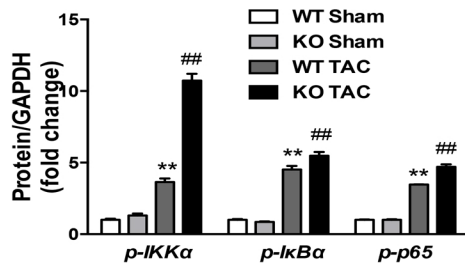

Supplement: Supplementary file 4 — Fig S4 [file JCMM-24-10946-s004.pdf]
