# Supplementary material for: Ubiquitin‐specific protease 19 blunts pathological cardiac hypertrophy via inhibition of the TAK1‐dependent pathway
Source: J Cell Mol Med. 2020 Aug 14;24(18):10946–57. doi: 10.1111/jcmm.15724 (PMC7521154; doi:10.1111/jcmm.15724)
Supplement: Supplementary file 5 — Supplementary Material [file JCMM-24-10946-s005.docx]

Figure S1. The link between SIAH2 and USP19 in hypertrophic cardiomyocytes. A. the levels of USP19 mRNA expression was assessed in hearts in sham and TAC groups (n=4 mice per experimental group). B. the levels of USP19 mRNA expression was assessed in cardiomyocytes in PBS- and PE-treated NRCMs (n=3 independent experiments). C. the levels of SIAH2 mRNA expression in hearts of sham and TAC groups (n=4 mice per experimental group). D. Left, Western blots showing the USP19 expression in NRCMs in AdFlag-transfected/PBS, AdFlag-transfected/PE, AdFlag-tagged SIAH2 transfected/PE groups. Right, statistical analysis of USP19 protein (n=6 independent experiments). ** P＜0.01 vs AdFlag-transfected/PBS, ## P＜0.01 vs AdFlag-transfected/PE. E. Left, Western blots showing the USP19 expression in NRCMs in AdshRNA-transfected/PBS, AdshRNA-transfected/PE, AdshSIAH2 transfected/PE groups. Right, statistical analysis of USP19 protein (n=6 independent experiments). ** P＜0.01 vs AdshRNA/PBS, ## P＜0.01 vs AdshRNA/PE.

Figure S2. USP19-deficiency exacerbates heart hypertrophy and fibrosis in response to angiotensin II (AngII) in vivo. A, B, C, The ratios of heart weight/bodyweight (HW/BW; left), lung weight/bodyweight (LW/BW; middle) and heart weight/tibia length (HW/TL, right) were assessed in wild-type (WT) or KO groups subjected to saline or AngII infusion (n=10 mice per experimental group). D, E, F, G, Echocardiographic assessment (n=4 mice per experimental group) of left ventricular end-diastolic diameter (LVEDd, Left), left ventricular end-systolic diameter (LVESd, Middle left), fractional shortening (FS, middle right), ejection fraction (EF, right). H, representative images of the maximum section of the heart and cardiomyocytes (scale bar, 25μm for haematoxylin-eosin (H&E) staining) in indicated groups (left), statistical analysis of the cross-sectional areas of cardiomyocytes in indicated groups (n=80 cells per experimental groups, right). I, representative images of the cardiac fibrosis in indicated groups (scale bar, 50μm for picrosirius red staining, left), Statistical results for fibrotic areas in different groups (n=4, right). **P＜0.01 or *P＜0.05 vs WT/saline; ##P＜0.01 or #P＜0.05 vs WT/AngII.

Figure S3. Total levels of protein detected using BCA assay in NRCRMs. A. Statistical analysis of USP19 protein quantitation in PBS- or PE-treated AdshRNA and AdshUSP19 groups (n=3 per group). *P＜0.05 vs AdshRNA/PBS, #P＜0.05 vs AdshRNA/PE. B. Statistical analysis of USP19 protein quantitation in PBS- or PE-treated AdGFP and AdUSP19 groups (n=3 per group). **P＜0.01 vs AdGFP/PBS, #P＜0.05 vs AdGFP/PE.

Figure S4. A. Representative fluorescence imaging carried out with PBS or PE stimulation in NRCMs using confocal microscope to detect USP19 content and cellular distribution. B. Quantitative results of the relative mRNA levels of TNF-α, IL-1β in WT or USP19-knockout mouse treated with sham or TAC operation (n=3 independent experiments). C, Above, Western blots showing the phosphorylation and total protein levels of IKKα, IκBα and p65 in WT or USP19-knockout mouse treated with sham or TAC operation. Bottom, Statistical analysis of the phosphorylation levels of IKKα, IκBα and p65 proteins normalized to GAPDH (n=6 independent experiments). *P＜0.05 or **P＜0.01 vs KO sham, ##P＜0.01 vs WT TAC.
